# Road Curb Detection: A Historical Survey

**DOI:** 10.3390/s21216952

**Published:** 2021-10-20

**Authors:** Lucero M. Romero, Jose A. Guerrero, Gerardo Romero

**Affiliations:** 1 Electronics Department at U.A.M. Reynosa-Rodhe, Universidad Autónoma de Tamaulipas, Reynosa 88779, Mexico; a2133728003@alumnos.uat.edu.mx; 2 Institut Pascal, Université Clermont Auvergne, CNRS, SIGMA Clermont, F-63000 Clermont-Ferrand, France; jguerrer@ieee.org

**Keywords:** road curb detection, feature-based curb detection, classification-based curb detection

## Abstract

Curbs are used as physical markers to delimit roads and to redirect traffic into multiple directions (e.g., islands and roundabouts). Detection of road curbs is a fundamental task for autonomous vehicle navigation in urban environments. Since almost two decades, solutions that use various types of sensors, including vision, Light Detection and Ranging (LiDAR) sensors, among others, have emerged to address the curb detection problem. This survey elaborates on the advances of road curb detection problems, a research field that has grown over the last two decades and continues to be the ground for new theoretical and applied developments. We identify the tasks involved in the road curb detection methods and their applications on autonomous vehicle navigation and advanced driver assistance system (ADAS). Finally, we present an analysis on the similarities and differences of the wide variety of contributions.

## 1. Introduction

**Figure 1:**
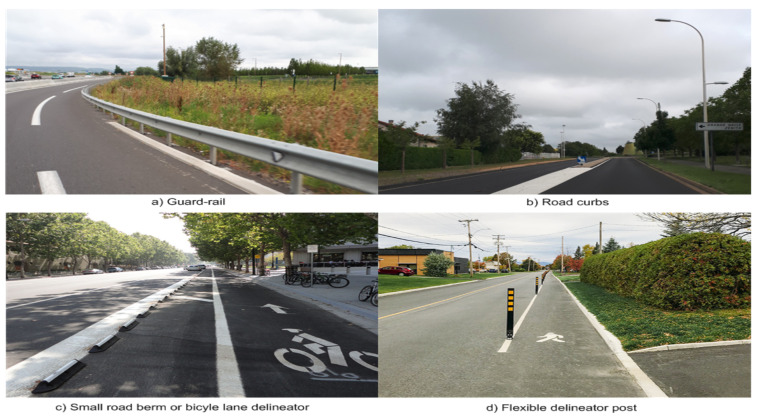
Road borders: (**a**) Guard rail on highway access road, (**b**) road curbs on urban environment, (**c**) small road berms used as bicycle lane delineators, and (**d**) multipurpose flexible delineator post.

Over the last two decades, the road curb detection problem has attracted the attention of research teams around the world. The technological advances leading to new generations of sensors have spurred the interest in the detection of road curbs. Road curb detection has been studied as a natural extension of the road boundary detection problem. Depending on the type of road (rural, urban, etc.), the road limits are defined using various structures, for example, guard rails, berms, road curbs, among others, see Figure 1.

**Figure 2:**
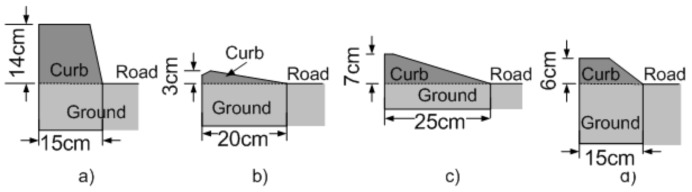
Types of road curbs: (**a**) Road-sidewalk, (**b**) island, (**c**) parking entry, (**d**) low height road-sidewalk, [1].

In urban environments, curbs are the most common structure used to delimit the navigable area for vehicles without doubt, they might take different shapes and sizes, as shown in Figure 2.

The accurate detection of these curbs or delimiters used in autonomous vehicles is useful for various tasks, e.g., path planning, path following, vehicle localization, and parking tasks to mention a few. Curb detection in combination with the former tasks allows to guarantee the integrity of the vehicle. Failing to detect small road curbs might result in damage of the vehicle’s suspension and/or vehicle roll-over situations. It is clear that curb detection is a fundamental requirement for autonomous vehicles’ safe navigation. Advanced driver assistance system (ADAS) is another important application for road curb detection methods since they provide the driver with information when the vehicle risks getting off the road, lane departure alerts, etc.

### Road Curb Detection Chronology

**Figure 3:**
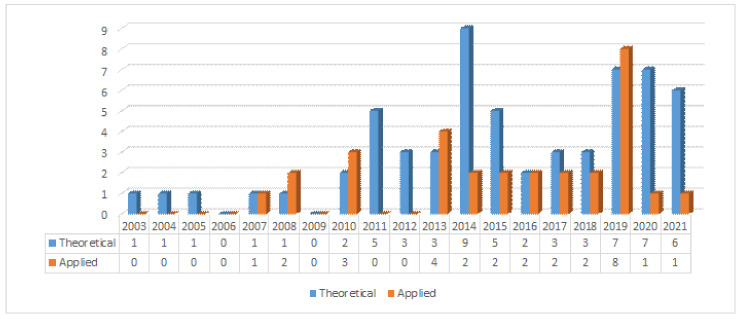
Road curb detection publications over time. Publications are organized into Theory (contributed method) and Applications.

Early road curb detection methods were based on vision systems and LiDAR sensors. In the early 2000s, road curbs were detected using histograms and Kalman filters, see [2,3]. By the mid-2000s, the Hough transform was introduced on LiDAR 2D data under the assumption that the terrain is flat [4,5,6]. Later on, stereo vision systems were used to detect road curbs on dense 3D data [6,7,8]. Stereo vision-based methods introduced the idea of using a Digital Elevation Map (DEM) combined with classical edge detection methods and the Hough transform. By the end of the 2000s, omnidirectional vision cameras were used, for instance, to monitor intersection traffic and road detection. A new generation of LiDAR sensor capable of scanning the environment at 360° also became commercially available by the end of the 2000s. A wide variety of methods have been proposed using such LiDAR sensors. For instance, the authors of [9] mimic a catadioptrical camera using a LiDAR3D by projecting LiDAR’s range measurements into an omnidirectional image in which road boundaries are searched in off-road conditions. Since stereo vision and LiDAR sensors provide either dense or sparse 3D data, the use of Digital Elevation Map became a common practice in the road curb detection problem [6,8,9,10,11,12,13,14]. To search for road curbs on sparse 3D data provided by LiDAR 3D sensors, ground segmentation [15,16,17,18] and feature extraction [15,17,19,20,21,22,23,24,25,26,27,28,29,30] have been widely discussed over the last decade. High-level representation or model of detected road curbs have been proposed in the last decade using polynomial curves [8,10,31], and splines [9,32]. In the recent years, new techniques such as neural networks [21,28,33,34,35], and deep learning have been used [35] to solve road curb detection as a classification problem. Without a doubt, there is a growing interest in the road curb detection problem, as it can be observed in Figure 3.

This paper is structured as follows: Section 2 presents the general curb detection methodology, which is common to most of the existing literature. The applications in which road curb detection is very useful are shown in Section 3. Finally, in Section 4, we present future challenges and trends in the detection of road curbs.

## 2. Curb Detection Methodology

**Figure 4:**
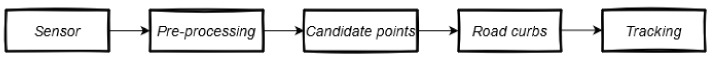
Blocks Diagram for the road curb detection.

Road curbs in most urban or semi-urban environments are present on both sides of the road, they are very small objects from which we can extract geometric and appearance attributes. Curb detection methods are often based on multi-stage algorithms or pipelines, as shown in Figure 4. They are discussed in the following sections.

### 2.1. Data Acquisition

**Figure 5:**
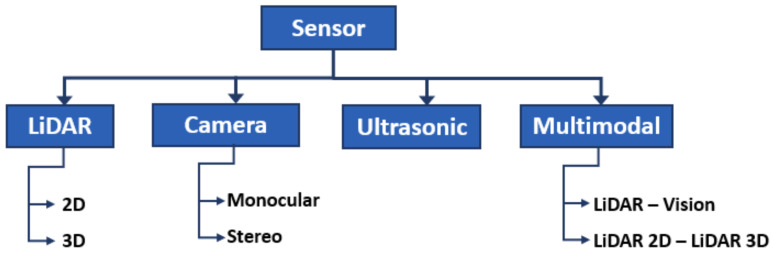
Types of sensors for data acquisition.

Due to the interest in solving the curb detection problem, different methods have been developed over the years. They can be classified using several criteria, one of which is by the type of sensor used for data acquisition, for instance, vision (monocular, stereo, omnidirectional), LiDAR (2D or 3D), ultrasonic, or a combination of these sensors (multi-modal detection), as shown in Figure 5.

#### 2.1.1. Vision-Based

**Figure 6:**
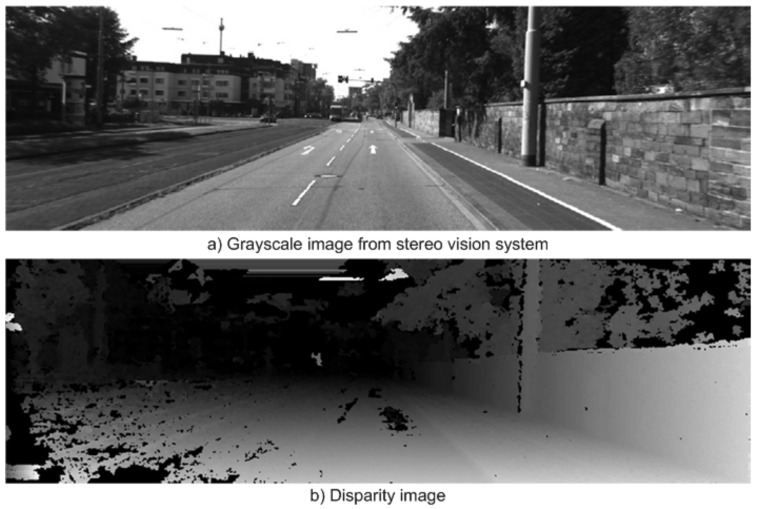
Curb detection using stereo vision system [36].

Cameras are passive sensors that have been widely used in autonomous vehicles due to the high information content they provide, low operating power, and low costs. However, they have certain well-known disadvantages, for example, shadows, complex navigable environments, poor lighting and bad weather. Thus, it is often difficult to extract features from the road curbs on vision-based perception systems. To ease the detection of road curbs, a laser line stripper and a camera have been used in [2]. Road curb detection has since been improved using a stereo vision system [6,10,36], which allows to obtain an estimation of the depth in the image through the disparity map, see Figure 6. Such systems allow the creation of a digital elevation map in which road curbs are found.

#### 2.1.2. LiDAR-Based

**Figure 7:**
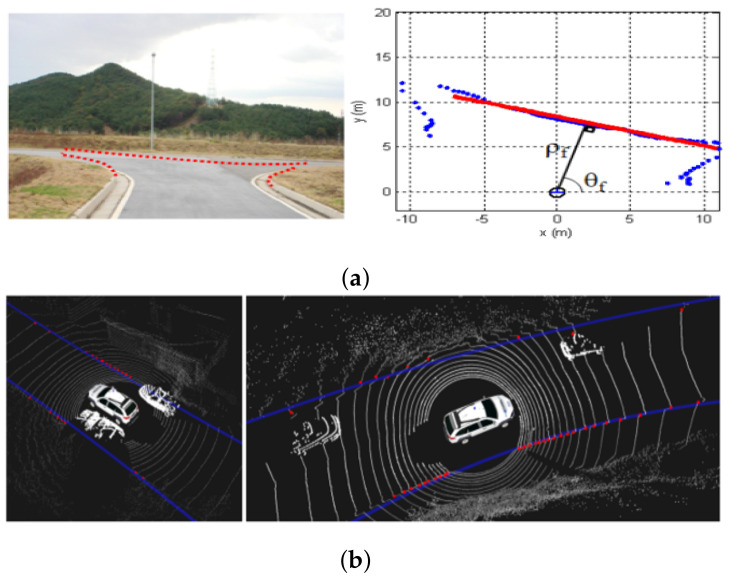
Road curb using LiDAR sensors: (**a**) LiDAR 2D [27]. (**b**) LiDAR 3D [39].

Active sensors such as LiDAR and MMWR (radar) are capable of providing 3D information of the environment. However, interference between multiple LiDAR (or radar) sensors is a known problem when they are close to each other. In particular, radar sensors tend to have low resolutions and slow scan speeds. On the other hand, recent advances in technology allow LiDAR sensors to operate at higher frequencies than 10 Hz. Laser sensors have a larger field of view (FoV) than vision-based perception systems and can cover moderate distances around 100 m and up to 220 m. They are sensitive to some atmospheric disturbances, such as rain, fog, dust clouds, etc. LiDAR sensors in automotive applications have been applied for obstacle detection and determination of road limits by curb detection. Often the road curb detection problem in LiDAR2D data [3,12,27], among others, is translated into finding the extreme points of a line that traverses the road. LiDAR3D sensors have been widely used to solve the road curb detection problem [17,22,37], as well. They provide both sparse and dense point clouds, depending on the sensor’s number of laser beams, of the environment as shown in Figure 7. In most cases, the point cloud goes through a pre-processing stage that extracts the ground points where curbs are more likely to be found. LiDAR point clouds are usually organized in voxel grids, elevation maps, or occupancy maps [9,13,19,38].

#### 2.1.3. Ultrasonic-Based

**Figure 8:**
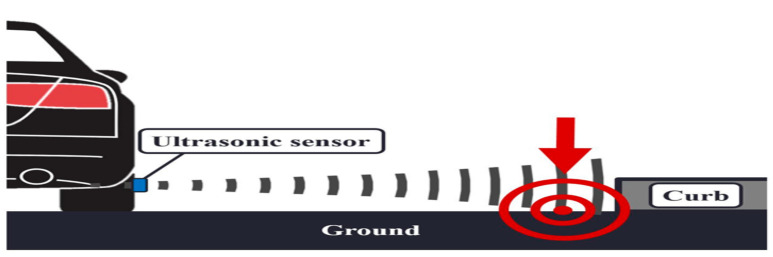
Curb detection using ultrasonic sensors [40].

Ultrasonic sensors are active sensors that detect the echo from the closest obstacle, see Figure 8. Their detection range is often limited from several centimeters up to few meters (often <10 m). Recently, attempts to detect road curbs using ultrasonic sensors have been made [40]. The main disadvantages are accuracy and the detection range. It should be noticed that results reported in the literature show that it is possible to detect road curbs using ultrasonic sensors rather than showing an improvement on the curb detection problem.

#### 2.1.4. Multi-Modal

**Figure 9:**
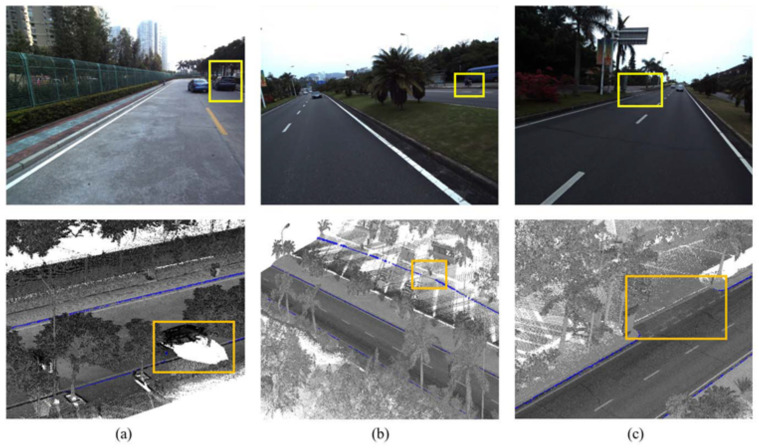
Curb detection using vision and LiDAR sensors: (**a**) Incomplete boundaries due to occlusions, (**b**) Worn road curbs, (**c**) A connection area between bikeways and roadways [42].

Data fusion using multiple sensors has been used to improve detection accuracy and precision. The multi-modal curb detection methods have been boosted by the convolutional neural network method. An early multi-modal curb detection method was introduced in [30] where LiDAR and images are fused to create a depth image of the scene. Then, the depth image is used for curb detection. Data fusion of image data and multiple LiDAR sensors was presented in [33,41]. Data are then fed into classification algorithms to deal with the curb detection problem, see Figure 9.

A synthesis of the contributions based on the sensory mode is shown in Table 1.

### 2.2. Pre-Processing

In this section, we identify and discuss the various pre-processing methods used in road curb detection algorithms for noise removal on raw sensor data. It is well known that data provided by sensors are often affected by different uncertainty and/or noise sources. For instance, in [6], the accuracy of the elevation at a given depth *Z* on a stereo vision-based system depends on various parameters, such as baseline *B*, focal distance *F*, disparity uncertainty $D_{e}$:

(1) $$Z_{e}=\left|\frac{Z^{2}D_{e}}{BF-D_{e}Z}\right|$$

LiDAR range measurements can be affected by multi-path range measurements, for example, object’s reflective properties and geometry. Thus, most existing curb detection methods involve the use of some type of noise reduction method on digital images and/or LiDAR point clouds. Noise removal techniques used in curb detection systems mainly focus on removing aberrant and smoothing sensory data.

Smoothing filters have been used in both image and LiDAR point clouds. Examples of smoothing filters are: (a) CS median filter used in [4] to remove noise from LiDAR data while preserving the intensity of the edges (curbs) and (b) Gaussian filter that has been used on depth images in [30]. The Gaussian filter has also been applied to the elevation on raw point clouds in [62].

Temporal filtering has been used mainly in the creation of a Digital Elevation Map. To increase DEM accuracy, consistency and to reduce noise, a temporal filter was used in [7]. In [10,32,41,63], temporal filtering has been used to address the sparsity of curb points detected in consecutive frames. This is done after removing the outliers from detected road curbs and consists of accumulating points in the current frame from a fixed sequence of past frames at the expense of the localization error.

Aberrant range measurements and outlier removal has been used in [1,3], etc. The authors of [3] used an extended Kalman filter (EKF) to simultaneously filter random errors, remove outliers, and segment LiDAR measurements into straight line segments. A sensor’s geometrical model has been used in [1] to remove aberrant range measurements on a LiDAR 3D sensor.

### 2.3. Data Representation

This section discusses the most common data representation structures used by road curb detection algorithms: surface modeling through elevation maps and point cloud segmentation and voxel grids.

#### 2.3.1. Digital Elevation Map

**Figure 10:**
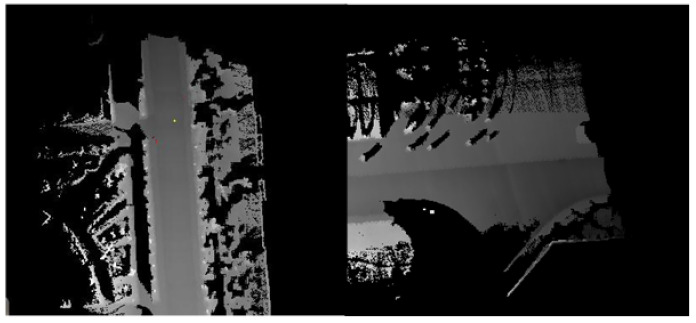
Digital Elevation Map from LiDAR data [13].

The use of the digital elevation map (DEM) is generally applied to represent the terrain’s geometry. It is computed from the 3D data obtained from the sensors into a Cartesian DEM [6,8,10,12,13,14,28,64] or polar DEM [9]. A Digital Elevation Map is a Cartesian grid made up of cells, where each cell contains an elevation value and it is often treated as a gray-scale image, see Figure 10. The elevation value in every DEM cell is often computed using the mean, the median, or the histogram elevation value of the measurements within that cell or bin. As discussed in [11], a DEM can be used to determine plane segments that can be considered as drivable regions. The idea of using a Polar DEM, discussed in [9], consists in generating an omnidirectional elevation image from a 360° LiDAR3D sensor. Curb detection, using edge detection algorithms, takes place on the resulting polar DEM. In general, the DEM is used to extract curb candidate points by using well-known edge detection methods such as Canny and Sobel [12,13,14,28,64].

#### 2.3.2. Point Clouds

Point clouds are a discrete representation of the environment in 3D that usually contains thousands of points. In order to locate road curbs efficiently, it is important to focus the search on the ground points. The point cloud segmentation process is used to generate two new point clouds: ground and non-ground. This process has been done using various methods, including directional normal, plane fitting, etc. Using point cloud segmentation allows to focus the road curb search on the ground instead of the whole scene. By doing so, the pre-processing time is largely reduced allowing for real-time curb detection.

#### 2.3.3. Voxel Grids

**Figure 11:**
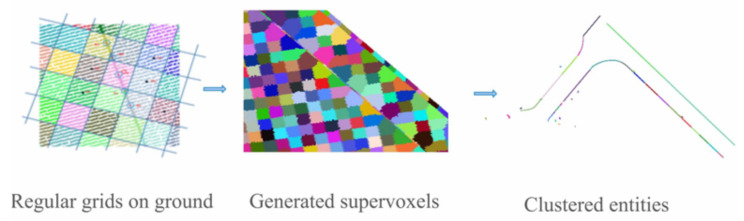
Supervoxels used in [56] for road curb candidate extraction.

Voxel grids are a discrete 3D representation of the space where voxels are 3D cubes whose dimensions depend on the selected voxel resolution. They have been used in [19] to speedup the ground segmentation process, the point clouds provided by a LiDAR 3D sensor in [19] are organized in a voxel grid. Then, ground segmentation is obtained by using the elevation difference between voxels. Finally, the resulting voxel grid is transformed into an elevation map where the road curb detection process takes place. The authors of [56] used supervoxels to extract road curb candidates from ground-segmented point clouds. Supervoxel candidates are then processed through clustering and other refinements to generate vectorized road boundaries, see Figure 11.

### 2.4. Ground Segmentation

**Figure 12:**
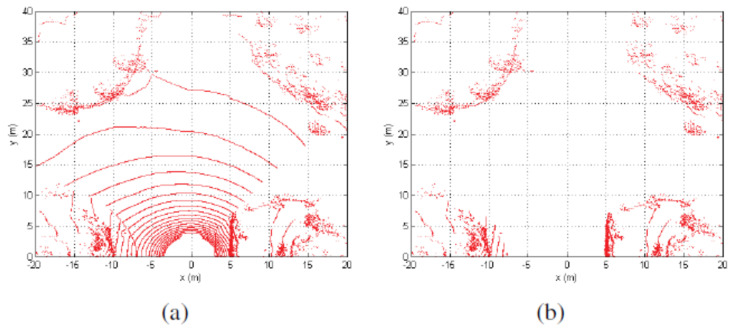
Point cloud segmentation using geometric constraints [50]. (**a**) raw data distribution under the vertical view; (**b**) filtered data distribution under the vertical view.

Ground segmentation is often computed under the assumption that the ground is flat. A point cloud segmentation is performed, in [16], based on the relative sizes of the normalized eigenvalues and the direction normal to the surface. Another idea that has been explored on the ground segmentation process is the use of a plane fitting on a DEM [64]. Although a plane fitting on LiDAR3D point clouds efficiently extracts ground points from a point cloud, there are situations in which the plane fitting will undoubtedly fail. For instance, when driving near large and tall walls, most of the LiDAR3D points will lay on the wall instead of the ground. Under the assumption of flat ground [17], the authors of [52] use the projection of LiDAR3D points into a plane. Points are selected as ground points if they form concentric circles. The drawback of the point cloud segmentation proposed in [17] is that roads follow the terrain geometry leading to multiple situations in which projecting LiDAR points into a plane might fail. In [50,53], the problem of road curb detection at intersections is solved using a beam (sliding or double layer) method, see Figure 12. An improvement on ground segmentation has been presented in [1] where the sensor’s geometric model is used to segment LiDAR3D point clouds into ground and non-ground.

The semantic segmentation by pixels is performed using neural networks in [28] where a CRF refines the results obtained by a CNN called SegNet. Other methods using convolutional neural networks to segment point clouds by assigning a label provided by the CNN to each point can be found in [33,41].

### 2.5. Feature Extraction/Attribute Extraction

To detect road curbs on both image and point clouds, a wide variety of features have been used over the last two decades. Most of these features are geometric features computed on point clouds. It is worth mentioning that in order to speed-up the road curb detection process, feature extraction takes place on ground-segmented data.

#### 2.5.1. Height Step

**Figure 13:**
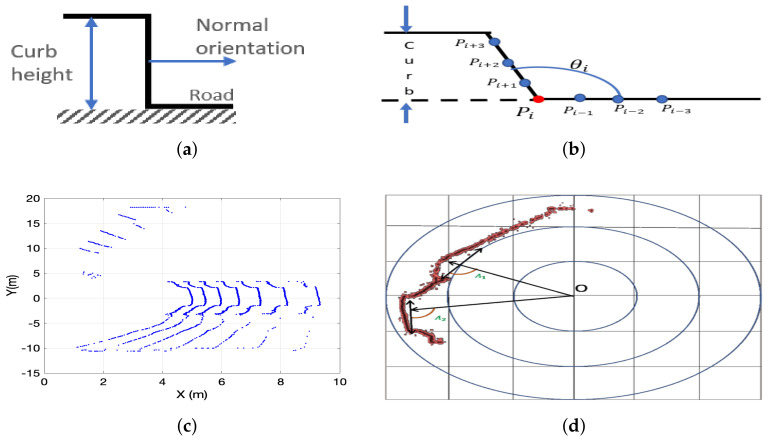
Road curb geometric features: (**a**) Curb height step and normal orientation. (**b**) Slope angle formed by the road surface and the curb. (**c**) LiDAR 3D conic section compression. (**d**) Tangential angle.

It is one of the most commonly used features on point clouds provided by stereo vision systems and LiDAR sensors, see Figure 13a. It was used in [4] to determine the curb candidates on range images. These curb candidates are then filtered using mathematical morphology operations such as morphological open and close. An analysis of LiDAR2D range measurements in search for road curbs as the closest prominent height step was presented in [26] while the height difference on disparity images to detect curbs on images from a stereo vision system was used in [60]. In [46], a height difference is computed using an estimation of the height difference which is computed using a trapezoidal rule of integration. Height difference on a super voxel grid was used in [56]. A similar feature has been applied on the projected surface or plane where the horizontal distance between consecutive points is computed as in [65]. In [25], the average and the variance of the normalized height difference are used as features to determine which LiDAR3D points belong to the surface and those considered as curb candidates. This feature has been applied to LiDAR3D data in [17,19,22,24,33,41,50,51,55,59,62,65].

#### 2.5.2. Height Gradient

Height gradient has been used mainly in DEM-based road curb detection methods. It was first used in [6] where a Sobel filter is used on an elevation map of the road before searching for road curbs. In [19], LiDAR points are stored in voxels from which the elevation gradient is computed. The voxel grid is transformed into a 2D elevation map, which they convolve with a Gaussian filter. A 3 × 3 Sobel operator is used to obtain the gradient in the horizontal and vertical directions. Height difference or gradient is computed on a DEM in [12,13,14,30]. The well-known Canny edge detector was used in [64] as a preliminary step for curb candidate extraction. The gradient has been applied in [57] to density occupancy maps obtained from LiDARD data. In addition, the authors of [65,66] use the gradient of gray-scale images as appearance features for classification-based curb detection.

#### 2.5.3. Normal Orientation

This feature describes the orientation of a small surface patch which in turn allows determining the location of a curb by searching for an abrupt change in the normal orientation, see Figure 13a. Normal orientation has been used on voxel maps, dense point clouds, depth images, etc. In [19], the surface normal is computed using the Principal Component Analysis method on every ground point and all points located on the same voxel are considered its neighbors. Normal orientation on a feature map obtained from dense point clouds has been used in [45]. Similarly, normals have been computed on depth images in [30] while surface normals on the 3D information obtained from a disparity map has been discussed in [65]. In [59], the normal orientation is used to verify that curb candidate points separate two horizontal planes representing the sidewalk and the road.

#### 2.5.4. Slope Angle

In [43], a tangent angle feature is defined as the angle formed by two vectors as shown in Figure 13b. The angle is defined as [43]:

(2) $$\theta_{i}=cos^{-1}\left(\frac{V_{i}^{R}V_{i}^{L}}{|V_{i}^{R}\left|\right|V_{i}^{L}|}\right)$$

Slope angle on DEM is used in [12,13]. Additionally, the authors of [55] compute the slope as the arctan of the elevation difference between consecutive LiDAR3D points.

#### 2.5.5. Conic Section Compression

Ring (circle) compression or LiDAR radius gradient-based method was introduced in [67] to detect curb-size obstacles. An extension of this method has been presented in [39]. This method estimates the radius of the ideal circle drawn by a 360° LiDAR 3D sensor on flat ground. Curbs produce compression on the ideal circles as shown in Figure 13c. Since ring compression uses a projection of LiDAR points on an ideal plane, it is not clear how effective this feature is on non-flat roads. This feature was used in [17,34,48]. In addition, it should be noticed that a 360° LiDAR 3D sensor, for instance, the Velodyne HDL32E LiDAR sensor, draws a conic section on a flat surface depending on the orientation of the sensor on top of the autonomous vehicle, see [1,33,41].

#### 2.5.6. Tangential Angle

In [17,52], the angle between its radial direction and its tangential direction is measured on LiDAR3D data projected onto the road surface, see Figure 13d. A similar approach was presented in [68], and used in [24,33,65], where the angle formed by two vectors are drawn from a given point $p_{i}$ is used as a feature for curb candidate extraction.

#### 2.5.7. Curvature

Surface curvature features on 3D point clouds and disparity images were used in [28,29,36,39,61]. These features are computed using the covariance matrix of *p* nearest neighbors.

#### 2.5.8. Smoothness

This feature was introduced in [69] and used in [22,24] to describe the smoothness around some point $p_{m,i}$.

(3) $$c=\frac{1}{|S|∥p_{m,i}∥}\cdot ∥\sum_{p_{m,j}\in S,j\neq i}\left(p_{m,i}-p_{m,j}\right)∥$$

where S is the cardinality of the set *S*. Larger values of *c* might be considered as salient points depending on a given threshold $T_{0}$. Values $c<T_{0}$ are considered as smooth points, i.e., they belong to plane surfaces.

#### 2.5.9. Smooth Arc Length

**Figure 14:**
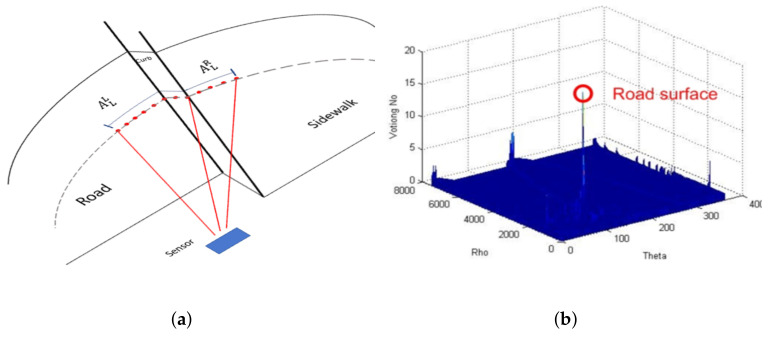
Geometric features: (**a**) Smooth arc length feature on LiDAR pointclouds. (**b**) Accumulator of the Hough transform used for road curb detection presented in [5].

In [22], under the assumption that road and sidewalks are smooth surfaces, this feature describes the arc length, described by a LiDAR 3D sensor, in a local window. This feature determines the arc length on each side of a given point $p_{m,j}$. At least one side of the point $p_{m,j}$ should have a smooth arc length, see Figure 14a.

#### 2.5.10. Hough Transform

Also called the line identification method, it is used to detect curbs [4] in the images once a set of candidate points has been extracted. In [5], they use the Hough transform to extract the longest straight lines from the data provided by a 2D LiDAR, see Figure 14b. These lines are considered the road surface and the curbs are located at their limits. Points lying on a small ρ distance are selected as part of the longest straight line. [6] uses the Hough accumulator in conjunction with a validation function to reject false positives. The process starts by detecting the edges in an elevation map; once they have a set of these edges, they calculate “relevant” lines through the Hough accumulator, which is used with a set of criteria that aid in detecting the road curbs. Other methods that use Hough transform to extract curb candidate points are reported in [12,13,22,64,66].

#### 2.5.11. Line Segment Analysis

**Figure 15:**
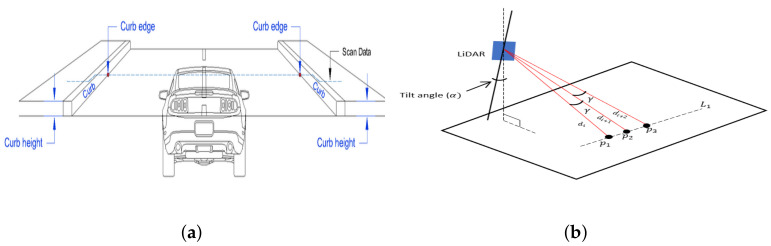
Line segment analysis proposed in [3] for road curb detection. (**a**) Line segment analysis. (**b**) Laser data points on road surface.

In [3], a Kalman filter is used to identify straight lines from LiDAR2D data. Since road curbs are parallel to the road, line segments whose orientation lies on a window defined by the minimum radius of the road, road width, and a look-ahead distance are extracted, see Figure 15. Road width is assumed to be known through the use of GPS and digital maps. The authors of [27] define the curbs as lines whose orientation is nearly perpendicular to the line(s) that describe the road whose width is supposed to be known.

#### 2.5.12. Elevation Histogram

To extract the road curb location, the authors of [2] use a histogram built from distance measurements obtained with a camera and a line stripper vision system.

#### 2.5.13. Laser Reflectance

Laser reflectance is a feature that is rarely exploited. However, it allows to determine the type of material an object is made of. It has been used in [1] to improve curb detection in ground-segmented points clouds which contain range measurements from the asphalt, road markers, and concrete curbs. The spectral analysis presented in [70] shows that reflectance is a good discriminant for these materials.

#### 2.5.14. Integral Laser Point

In [37], the authors introduced the integral Laser Point (ILP) features on each scanning line of a Velodyne HDL64 LiDAR3D sensor. This feature is used to compute other geometrical curb features such as height differences, etc.

#### 2.5.15. Radon Transform

**Figure 16:**
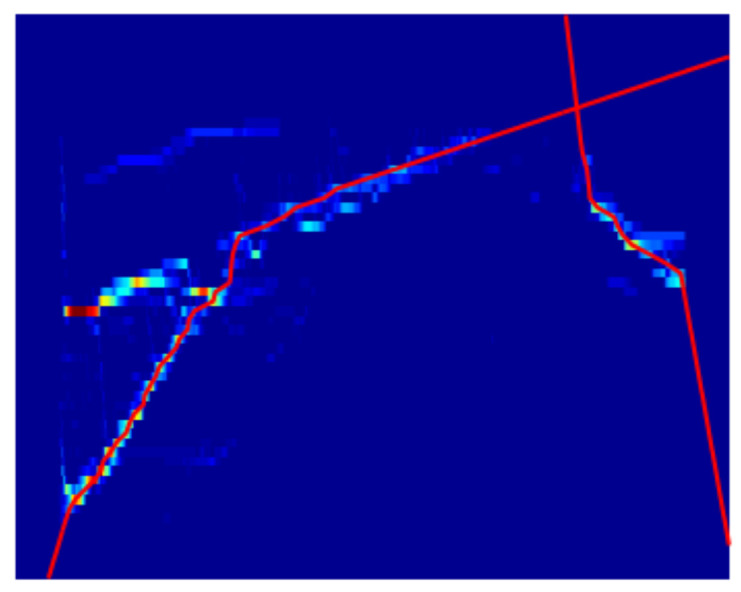
Radon transform application [71].

In [71], the authors propose to transform the road curb detection problem on a stereo vision system by using the Radon transform of the histogram of the disparity map, see Figure 16. Notice that, as discussed in [72], the Radon Transform and the Hough Transform are closely related although they are not the same. Then, the road curb detection problem is reduced to the problem of peak detection. To deal with curved curbs, a Viterbi search space is used.

#### 2.5.16. Discrete Haar Wavelet

This features was used in [15] to extract features from multiple LiDAR sensors. This method generates a series of waveforms that convolved with every LiDAR scan provide the terrain’s slope over small windows of data. The coefficients provided by the Haar wavelet transform are then used to label points as road or non-road. Using the Haar wavelet might result in a large number of false positives and post-processing is required to remove them.

#### 2.5.17. Texture

Features, such as the average, median, variance, and sparsity are computed on a disparity texture map which are then used for classification-based curb detection using a SVM classifier [65].

#### 2.5.18. Histogram of Oriented Gradients (HOG)

A histogram of oriented gradients was used in [66] to extract attributes to be used on machine learning algorithms for curb detection.

#### 2.5.19. Bayesian Filter

A Bayesian filter approach has been used in [58]. First LiDAR data are split into uniform sections and, then, they are compared with a predefined curb model with fixed height and width. The probability that a LiDAR data section belongs to a curb is computed using

$$p\left(cell_{k}\right)=\prod_{i=0}^{n}exp^{-\frac{e_{i}^{2}}{2\sigma^{2}}}$$

#### 2.5.20. Local Binary Patterns

Local binary patterns (LBP) have been used in [20] to extract features from a monocular vision system for classification purposes into four classes: road, non-road, curb, and soft shoulders. Although good results have been reported using LBPs, their performance seems to be guaranteed in good weather conditions and daytime only.

### 2.6. Road Curb Detection

Curb detection methods can be organized into two categories: thresholding and classification. Thresholding usually is applied to multiple geometrical and/or appearance features while classification-based methods rely on well-known machine learning techniques such as support vector machines and neural networks.

#### 2.6.1. Thresholding

In [2], road curbs are detected using thresholding on a histogram of the measurements obtained using a camera and a LiDAR line stripper. In addition, in [5], a threshold is used on both the distance and the slope of consecutive points belonging to the longest straight line (road). The authors of [16] use this method on elevation gradient and surface normals of point clouds which result from a ground-non-ground segmentation. Another method is presented in [37] where road curb detection is divided into two stages. In the first stage, it projects the 3D LiDAR data into the *x*-*y* plane and, then, it applies a line fitting strategy on a sliding window using *N* points and computes the maximal intensity difference to locate road curbs. This is done by applying thresholding on the detected line slope, the fitting error, and the intensity difference. The second stage also exploits the ILP features to compute the average elevation on a sliding window. Road candidate curb points are detected using a threshold on the average elevation. The use of ILP features allows them to reduce the complexity of the algorithm. Other methods that use thresholding on geometric and/or appearance features are reported in [3,4,7,16,17,24,25,26,36,39,46,48,49,52,71,73].

#### 2.6.2. Classification

Various learning frameworks have been used to detect curbs ranging from Conditional Random Fields, Gaussian Process Regression, SVM to Convolutional Neural Networks.

The CRF framework was used in [11] to label DEM cells into regions which allows to segment the map into drivable regions. Similarly, the authors of [29] use the conditional random field method to classify image pixels into curb or non-curb. In [13,22], a Gaussian process regression (GPR) is used to detect road curbs. A multi-layer perceptron neural network has been used in [20] to detect road curbs from LBP histograms which provide the probability of a pixel to belong to the curb class. Pixels are assigned to a class using a threshold which is often defined experimentally. The authors of [21] feed textural features to a neural network to classify a region of interest (ROI) as road or non-road on a single front camera.

The problem of road curb ramp detection using a multi-stage detection process is discussed in [74]. The first stage is to use a deformable part model (DPM) method [75] to detect road curb ramps. Then, non-maxima suppression is used to remove outliers, and finally, the output of the outlier removal stage is fed to an SVM classifier. The SVM framework has been used in [65,66] to detect road curbs from a trained dataset using 16 appearance and geometric feature descriptors. The authors of [31] detect road curbs using an Iterative End Point Fit segmentation and classification of segments and a hierarchical classification method.

A Convolutional Neural Network (CNN) called Segnet has been used in [28] to compute potentials that are combined with information from a DEM and curvature features in a Conditional Random Field whose output is used for curb extraction using a Canny edge detector. The authors of [33,41] use ERFNet on a multi-camera system to obtain semantic segmentation of the scene. Then, labels are assigned to LiDAR3D point clouds which are subsequently refined for curbs by using ROIs. Finally, ROIs are analyzed for curb refinement using geometrical features. Two different convolutional neural network architectures to extract road curbs from both image and LiDAR3D point clouds have been presented in [42] while [76] uses imitation learning to extract road curbs using convolutional neural networks on imagery datasets and [77] uses convolutional neural networks to detect road curbs using a computer vision system.

#### 2.6.3. Post-Processing

Post-processing is used to remove false positives after curb detection. In [15], false positives are removed by labeling closest candidate curb points to the vehicle as curb points. The authors of [39] use various filters to remove curb false positives obtained using a geometrical feature-based segmentation process. These filters test candidate curb points for steepness using a convolution mask; a distance filter is used to preserve only the closest obstacles and a regression filter is used to complete the removal of false positives. A clustering-based outlier removal has been applied in [24].

### 2.7. Tracking

The extended Kalman filter (EKF) has been widely used to track road curbs between frames or LiDAR scans, see [3,17,24,50,51,78]. In [44], a mobile robot builds a road curb map that is used for curb tracking and tracing. The curb edges on both sides of the road are used for mapping and tracking. The authors of [43] use a particular filter on a laser scanner for curb estimation and tracking. In [19], they also use a particular filter for curb tracking; they follow the ridge points that belong to the curb.

### 2.8. Road Curb Detection Methods over Time

Table 2 presents a brief description, advantages and limitations of the various methods that have somehow been a milestone in the development of road curb detection methods.

## 3. Applications

There a variety of applications of road curb detection in intelligent transportation systems and autonomous mobile robotics, such as curbs/obstacle mapping [79], road segmentation as pre-processing for road marking classification [18,49], automatic label generation for curb detection [59], road curb detection performance toolbox [80], etc.

To generate an obstacle map, [79] uses the road curb detection method proposed in [3] to include road boundaries on its obstacle map. The authors of [18,49] use road curb detection for road segmentation. Once road segmentation is completed, they focus on the road markings classification problem. A road curb detection performance toolbox has been presented in [80]. This tool is intended to be method- and sensor-independent and to help improving road curb detection parameter settings. The authors of [59] automatically generate labeled datasets including curbs detected using geometrical features such as elevation and surface normals. Ring compression curb features have been used in [34] to identify the road shape using an artificial neural network classifier. The authors of [55] use road curb detection for road surface extraction.

### 3.1. Localization

The authors of [81] use the road curb detection method presented in [50] for map-based autonomous vehicle localization. The localization process is based on the Iterative Closest Point (ICP) method that allows the estimation of the transformation *T* between the detected road curbs and the curbs provided by a digital map. The authors of [62] have used detected road curbs for Monte Carlo-based vehicle localization.

### 3.2. Curbs Mapping

**Figure 17:**
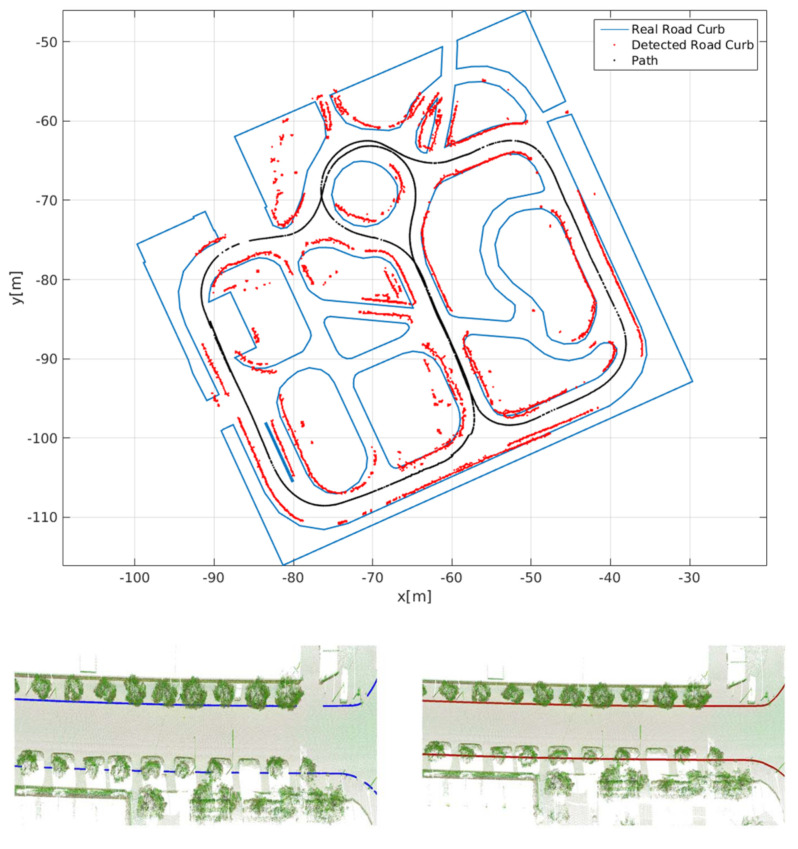
Curb mapping using a vision-based SLAM [1] and GPS [56].

Curb mapping has proven to be useful for autonomous vehicle localization and navigation, curb state monitoring, among other applications. Curb mapping in uneven urban roads has been presented in [1,62], etc. Detected curbs usually are aggregated in geolocalized maps. The accuracy of the curb map highly depends on the accuracy of the localization method. As shown in Figure 17, the difference between the generated curb map and the real geolocalized curb map is due to the error of the visual SLAM localization method used to create the curb map.

### 3.3. Road Curb Modeling

Various methods have been used to extract a high-level representation of curbs. They can be organized into polynomial, spline, regression.

- *Hough transform:* Hough transform is used in [12,13] to extract straight-line curbs from candidate curb points.
- *The polynomial fitting model:* To extract a mathematical model for detected curbs, the authors of [6] use lines, and in [7], a polyline model is used. In [19,37], road curbs are modeled using a parabola model. The authors of [8] propose to use a cubic polynomial instead of lines or polylines. A cubic polynomial allows to keep curvatures and their variations and they are in accordance with the clothoidal model for road lane boundaries. Cubic polynomial fitting has been used in [64]. Since the polynomial fitting problem is often overdetermined, they are solved using a least-squares method. A quadratic polynomial model has been used in [12,24,30] while [12] uses a cubic polynomial model to extract curved road curbs from candidate curb points.
- *Spline curb model:* A cubic spline has been used in [32] to improve curb modeling. The cubic spline interpolation is solved using least-squares minimization. The optimization follows an iterative approach until the optimal configuration of knots is selected. The increased complexity of using multiple iterations on the least square minimization is avoided by fitting the first polynomial inside its interval. The *k*th polynomial is fitted after it fulfills the continuity constraint. The authors of [56] use a cubic Bezier curve to fit extracted road curb points to model road curbs. The vectorized road curbs are then used to compute some road geometry parameters, such as driving free space, road width, road slope, horizontal curvature, etc.
- *Support vector regression (SVR):* Curb fitting using a support vector regression (SVR) was proposed in [54] that can handle high-dimensional and nonlinear problems. Results reported by [54] show that SVR improves the modeling of curved curbs with respect to linear [82] and cubic polynomial methods [8].

## 4. Future Challenges and Trends

In the recent years, road curb detection has attracted the attention of multiple research teams around the world. Road curb detection has become a fundamental problem for autonomous vehicle navigation in urban environments since they allow to detect the road boundaries and small obstacles. Road boundaries are then used by local path planning algorithms in autonomous vehicles. Curb detection on roundabouts can be seen as an extension of the classical road curb detection problem. 

## Figures and Tables

**Table 1:** Synthesis of contributions based on their perception mode.

Sensory Mode		References
Vision	Monocular	Aufrere et al. (2003) [2]
	Stereo	Oniga et al. (2007) [6]; Oniga et al. (2008) [7]; F. Oniga and S. Nedevschi (2010) [8]; Siegemund et al. (2010) [10]
LiDAR	2D	Wijesoma et al. (2004) [3]; Kim et al. (2007) [5]; Byun et al. (2010) [26]; Maye et al. (2012) [11]; Liu et al. (2013) [12], Liu et al. (2013) [13], Yu et al. (2015) [18], Lee et al. (2015) [23], Shin et al. (2010) [27], Byun et al. (2011) [43], Kim (2011) [44], Hervieu and Soheilian (2013) [45], Pollard et al. (2013) [46], Rejas et al. (2015) [47].
	3D	Wang et al. (2005) [4], Liu et al. (2015) [17], Chen et al. (2015) [22], Wang et al. (2019) [24], Liu et al. (2014) [25], Tan et al. (2014) [30], Hata et al. (2014) [34], Yao et al. (2012) [37], Hata and Wolf (2014) [39], Hata et al. (2014C) [48], Yu et al. (2015) [49], Zhang et al. (2015) [50], Zhang et al. (2015B) [51], Huang et al. (2017) [52], Zhang et al. (2018) [53], Zhao et al. (2019) [54], Zhu et al. (2019) [38], Guerrero et al. (2020) [1], Ye et al. (2020) [55], Mi et al. (2021) [56], D. Rato and V. Santo (2021) [57]
Ultrasonic		J. Rhee and J. Seo (2019) [40]
Multi-modal	Mono + Lidar	G. Zhao and J. Yuan (2012) [19], Tan et al. (2014) [30], H. Qureshi and R. Wizcorek (2019) [35], Fernandez et al. (2015) [36], Chun et al. (2010) [58], Kuhner et al. (2019) [59], Ma et al. (2021) [42]
	Fisheye + LiDAR	S. E. C. Goga and S. Nedevschi (2018) [33], Deac et al. (2019) [41]
	Stereo + Lidar	Tan et al. (2014) [30]; Fernandez et al. (2015) [36]
	Mono + stereo	T. Hu and T. Wu (2011) [60], Fernandez et al. (2017) [61]

**Table 2:** Curb detection methods.

Authors	Stages of the Method	Advantages	Limitations
Aufrere et al. (2003) [2]	(a) Laser triangulation, (b) Histogram, (c) Definition of the interest zone, (d) Canny edge detector.	Low cost and real-time detection.	It only detects curbs on one side of the road.
Wijesoma et al. (2004) [3]	(a) Road geometry using extended Kalman filter, (b) Straight line model, (c) Eigenvector technique, (d) Scatter matrix	Application of ladar for road-boundary determination through curb detection	Extensive processing to filter the lines corresponding to the curbs and arbitrarily select the threshold
Wang et al. (2005) [4]	(a) CS Median Filter, (b) Gradient, (c) Vertical differential operator, (d) Threshold, (e) Mathematical morphology, (f) Hough Transform	Using the CS median filter to intensify the edge of the gradient	To get accurate and correct curb information, they need to filter the data acquired from the sensor.
Kim et al. (2007) [5]	(a) Mapping, (b) Hough Transform	Detects curbs on both sides of the host vehicle	It requires information from various sensors.
Oniga et al. (2007) [6]	(a) DEM, (b) Edge detection, (c) Hough Transform, (d) Circular mask, (e) Median filter	Detect curbs having a height of at least 5 cm	With depth the 3D points reconstructed by stereo vision are sparser, this due to the perspective projection
Oniga et al. (2008) [7]	Extension of [6] which adds a Persistence Map (PM), threshold and RANSAC	Detects few false curbs and removes curved curbs	Not stable in detecting non-sharp curbs (so-called traversable)
Peterson et al. (2008) [15]	(a) Geometric features, (b) Segmentation (ground and non-ground clusters), (c) Wavelet (edge detector for curbs)	Curb Detection is performed to achieve its primary objective; road limits	They process a large amount of data
Stuckler et al. (2008) [9]	(a) DEM, (b) Linear interpolations, (c) Normalization, (d) Filter, (e) Spline	They use curbs to locate lanes in the road network	The model only considers the DARPA road network
Byun et al. (2010) [26]	Used threshold, based only a difference in lateral distance	Decrease calculation time by using ROI instead of full image	They consider that the road is flat and horizontal.
Chun et al. (2010) [58]	Bayesian filter	Application in autonomous navigation	They may get incorrect measurements, depending on the road conditions and ambient light
Oniga et al. (2010) [8]	(a) DEM, (b) RANSAC, (c) Cubic polynomial, (d) Temporal Filter	The DEM is suitable for real-time processing and it reduces the processing space	They use different methods to determine the coefficients of the cubic polynomial; depending on the number of points
Zhao et al. (2012) [19]	They build a 3D cubic voxel grid using the point cloud. They separate the ground points from the point cloud and use three spatial cues to extract the candidate curb points: the elevation difference, the gradient value, and the normal orientation.	Consider curb detection on both sides of the road	To obtain the evidence map, the previous 5 frames are required with the current one.
Hata et al. (2014) [34]	They segment the road from the measurements returned from a LiDAR (Velodyne HDL-32E) into two parts: curbs and road surface. This information is trained on an ANN multilayer perceptron to classify the road into eight different models. They use an obstacle detection method based on ring compression to detect curbs.	Contributes mainly by making possible the detection of several road geometries	They require extracting detailed information from the road to train an ANN for road geometry classification.
Sodhi et al. (2016) [28]	(a) Disparity, (b) SegNet-Segmentation, (c) Potential (curvature, height map), (d) Dense CRF	They combine semantic, geometric, etc. consistency signals by formulating dense CRF for long-range curb detection.	Combination of both appearance and 3D reasoning for a more accurate segmentation
Zhang et al. (2018) [53]	(a) Point cloud data, (b) Sensor calibration/plane-based filtering, (c) Sliding-beam segmentation/Segment-specific curb detection	Application of the sliding beam method to segment the road and detect various road shapes.	They filter both on road points and off road points
Wang et al. (2019) [24]	(a) Point cloud data/the position and pose changes of each frame, (b) Ground segmentation, (c) Density-based method, classifies left and right candidate points for road curb detection, (d) Candidate points filtering method is proposed which consists of distance filter and RANSAC filter, (e) Tracking, use an amplitude limiting Kalman filter to smooth the fluctuation of the road curb curve.	They only consider on-ground points for curb point extraction	They apply two filters: a distance filter and a RANSAC filter, to remove false points
J. Yu and Z. Yu (2021) [77]	(a) A mono-vision based lateral localization system, (b) CRoI module is proposed to obtain the curb region of interest, (c) semantic segmentation module based on the combination of U-Net and SCNN.	Efficient, low cost and less complex	The road scene classification module classifies road scenes only into three classes

## Data Availability

Not applicable.
